# Hidden ecological risks in reservoir sediments: grain-size dependent enrichment of toxic metals in the black sea basin

**DOI:** 10.1007/s10653-026-03281-x

**Published:** 2026-06-06

**Authors:** Koray Özşeker, Yahya Terzi, Coşkun Erüz, Neira Purwanty Ismail

**Affiliations:** 1 Institute of Marine Sciences and Technology, Karadeniz Technical University, Trabzon, Turkey; 2 Department of Fisheries Technology Engineering, Faculty of Marine Sciences, Karadeniz Technical University, Trabzon, Turkey; 3 Department of Marine Science and Engineering, Faculty of Marine Science, Karadeniz Technical University, Trabzon, Turkey

**Keywords:** Toxic metal, Granulometric controls, Fine particle enrichment, Ecological risk assessment, Reservoir ecosystems

## Abstract

Toxic metal accumulation in freshwater sediments poses a significant ecological threat, yet bulk sediment approaches often fail to account for particle-size–dependent variability in contamination and risk. This study investigates grain-size–controlled metal enrichment and seasonal dynamics in reservoir sediments from the northeastern Black Sea region (Türkiye). Surface sediments collected from the Borçka, Deriner, and Torul reservoirs were separated into four grain-size fractions (0.063–0.5 mm) and analyzed for Cu, Pb, Zn, Ni, and As. The finest fraction (< 0.063 mm) showed markedly elevated concentrations, reaching up to 409 µg/g for Cu, 156 µg/g for Pb, and 346 µg/g for Zn, substantially exceeding sediment quality guideline thresholds (PEL and ERM in several cases). Metal concentrations consistently decreased with increasing grain size (F1 > F2 > F3 > F4), confirming strong granulometric control. Seasonal patterns revealed higher concentrations in autumn, associated with enhanced fine particle deposition and reduced hydrodynamic conditions. Spatially, the Borçka reservoir exhibited the highest contamination levels, reflecting the influence of mining-derived inputs and anthropogenic pressures. Importantly, bulk sediment assessments may not fully capture localized metal enrichment associated with fine-grained fractions. These finding demonstrate that integrating grain-size–resolved analysis with ecological risk indices provides a more accurate and environmentally relevant framework for sediment assessment, with important implications for monitoring and management of reservoir ecosystems.

## Introduction

Sediments in freshwater ecosystems are not only passive sinks of contaminants but also dynamic regulators of ecological risk, particularly in systems subjected to intense anthropogenic pressure such as reservoirs (Muhammad, 2023; Özşeker & Terzi, 2025; Redwan & Elhaddad, 2022). Among various pollutants, toxic metals are of critical concern due to their persistence, non-biodegradability, and potential to accumulate in benthic environments, where they may be remobilized under changing physicochemical conditions (El-Sorogy et al., 2024; Ozseker, 2021). Reservoir systems, characterized by reduced hydrodynamic energy and enhanced sedimentation of fine particles, provide favorable conditions for the accumulation and long-term storage of metal contaminants. As a result, these environments act as both archives of historical pollution and active sources of secondary contamination, posing significant risks to aquatic ecosystems (Hanfi et al., 2020; Tang et al., 2024; Zeb et al., 2024).

Despite extensive research on metal contamination in aquatic sediments, most studies rely on bulk sediment analyses, overlooking the critical role of particle-size variability in controlling metal distribution and bioavailability (Özşeker et al., 2022a, 2022b, Zimmerli, et al., 2025). Fine-grained sediments, characterized by high surface area and strong affinity for organic and mineral phases, preferentially accumulate metals. However, studies integrating grain-size–rsolved analysis with ecological risk assessment remain limited, particularly in reservoir systems (Döndü et al., 2024; Mishra & Dubey, 2023; Muneer et al., 2022; Ozseker & Eruz, 2017). This limitation may reduce the ability to fully resolve contamination severity and associated ecological risks linked to fine-grained sediments.(Di Bella et al., 2024; Di et al., 2015; Özşeker et al., 2025a, 2025b).

The distribution of metals in sediments is influenced not only by their total concentrations but also by grain-size characteristics that control adsorption capacity and mobility (Gloaguen et al., 2021; Maslennikova et al., 2012; Özşeker et al., 2022a, 2022b). Parameters such as mineralogical composition, surface area, organic matter content, and cation exchange capacity collectively affect the extent to which metals bind to or are released from sediment particles (Al-Shihmani et al., 2024; Feng et al., 2026; Irshad et al., 2025; Xu et al., 2024). Numerous studies have shown that fine-grained sediments, especially clay and silt fractions smaller than 0.063 mm, exhibit a greater ability to retain metals due to their larger specific surface area, higher surface charge, and stronger affinity for organic and oxide phases (Gloaguen et al., 2021; Krumgalz et al., 1992; Maslennikova et al., 2012; Özşeker et al., 2022a, 2022b; Wang et al., 2023). In contrast, coarser particles (e.g., 0.25–0.5 mm) generally display lower adsorption capacities. Accordingly, examining metals across different grain-size fractions provides valuable insight into their accumulation and transport behavior within aquatic environments (Nguyen et al., 2025; Ontiveros-Cuadras et al., 2026; Subramanian et al., 2025).

Lake and reservoir ecosystems differ fundamentally from lotic or marine systems due to their relatively stagnant hydrodynamic conditions and stronger sediment–water interactions (Ozseker & Eruz, 2017; Özşeker, et al., 2025a, 2025b; Ranathunga et al., 2025; Tegegne et al., 2025). Reduced water movement facilitates the deposition of fine particles, while organic-rich sediment layers enhance the binding of metal ions (Lanzerstorfer, 2018; Özşeker et al., 2024). Periodic hypoxic or anoxic conditions in bottom waters can also alter redox potentials, leading to the remobilization of previously bound metals (Lu et al., 2022; Ye et al., 2024). Therefore, the distribution and bioavailability of metals in lacustrine sediments are regulated by complex interactions between physical and geochemical processes (Aleksander-Kwaterczak et al., 2021; Özşeker & Terzi, 2025). Importantly, the grain-size composition of sediments serves as an indicator of depositional energy, sediment provenance, and potential ecological risk.

To evaluate contamination severity and ecological implications, widely used geochemical and ecological indices such as Igeo, EF, TRI, and PERI are commonly applied. These indices provide a quantitative framework for linking metal enrichment with potential ecological risk (Duman & Eronat, 2026; Jahan & Strezov, 2018; Muhammad, 2023).

The Northeastern Black Sea region of Türkiye has recently experienced growing environmental stress due to hydroelectric dam construction, mining operations, and expanding urban and agricultural activities (Diktaş Bulut, 2023; Kekec et al., 2018; Özşeker & Erüz, 2011; Özşeker & Terzi, 2025; Özşeker et al., 2022a, 2022b). Reservoirs in this area serve as major hydrological control structures and energy sources, but also as depositional basins for land-derived pollutants (Özşeker et al., 2025a, 2025b; Ware et al., 2024; Xiao & Mohammaditab, 2024). Despite their ecological and economic significance, studies examining how sediment grain size influences heavy metal accumulation and ecological risk in these reservoirs remain limited, representing a key knowledge gap in both regional and global contexts (Kang et al., 2017; Maslennikova et al., 2012; Özşeker et al., 2022a, 2022b; Subramanian et al., 2025).

Despite growing interest in sediment-associated metal contamination, studies simultaneously addressing grain-size variability, seasonal dynamics, and ecological risk within a multi-reservoir framework remain scarce. Most existing research focuses on single systems or bulk sediment approaches, failing to capture how sediment characteristics interact with hydrodynamic conditions and anthropogenic inputs. Consequently, the ecological significance of fine sediment fractions is often overlooked, leading to incomplete or biased assessments of contamination and risk. To address this gap, the present study investigates the fraction-specific distribution of toxic metals across different grain-size classes (0.063, 0.125, 0.25, and 0.5 mm) in the surface sediments of the Borçka, Deriner, and Torul reservoirs in the northeastern Black Sea region of Türkiye. Sampling was conducted in spring and autumn to examine the effects of seasonal variability on metal accumulation, spatial distribution, and ecological risk.

The specific objectives of this study are to: (i) determine the spatial and seasonal variations of selected heavy metals in surface sediments of the Borçka, Deriner, and Torul reservoirs; (ii) evaluate the accumulation behavior of metals across different grain-size fractions (0.063–0.5 mm); (iii) explore the relationship between sediment texture and metal concentrations using statistical and ecological indices (Igeo, EF, TRI, and PERI); (iv) conduct a comparative assessment of the three reservoirs to reveal differences in contamination levels and source characteristics; (v) assess the influence of anthropogenic pressures and environmental factors on metal distribution patterns; and (vi) evaluate sediment quality and potential ecological risks within these aquatic systems.

It is hypothesized that fine sediment fractions not only exhibit higher metal concentrations but also disproportionately influence ecological risk patterns, potentially leading to incomplete interpretations when only bulk sediment characteristics are considered. Within this context, the present study represents one of the few comprehensive investigations in Türkiye examining heavy metal accumulation, seasonal variation, and source differentiation across multiple grain-size fractions in reservoir sediments. By integrating fraction-specific geochemical analysis with ecological risk assessment in a multi-reservoir framework, this research aims to provide additional insight into the physical controls governing metal enrichment and sediment-associated ecological processes in freshwater ecosystems.

## Material and methods

### Description of study area

The study was conducted in 2024 across three distinct lake ecosystems located in northeastern Türkiye: the Borçka, Deriner, and Torul Dam Lakes. Each site possesses unique environmental characteristics and varying degrees of human influence, providing a representative framework for assessing the dynamics of metal pollution within these aquatic ecosystems. Sampling was carried out during the spring and autumn seasons, enabling a comprehensive evaluation of seasonal variations in pollution levels under differing environmental conditions.

Borçka Dam Lake, located on the Çoruh River in Artvin province (Türkiye), was completed in 2005 as part of a large-scale hydroelectric development initiative aimed at enhancing regional energy production. Formed by a double-curvature concrete arch dam, the reservoir effectively utilizes the steep slopes and narrow valleys characteristic of the Çoruh Basin’s geomorphology. Several small tributaries and surface runoff channels discharge into the reservoir, particularly during periods of high precipitation, contributing increased loads of suspended sediments, nutrients, and trace metals that significantly influence its hydrological and geochemical dynamics. Among these, the Murgul Stream, draining an area with extensive mining activities, constitutes a major input pathway for metal-rich sediments and mine-derived contaminants. In addition to these natural and mining-related inputs, the catchment is subjected to various anthropogenic pressures. Upstream settlements, transportation corridors, agricultural lands, and small-scale industrial facilities introduce pollutants into the system through domestic wastewater, agricultural runoff, and erosion-driven material transport. Such inputs can adversely affect water quality, enhance metal accumulation, and impose varying levels of ecological stress on the reservoir ecosystem. Beyond its primary function in hydroelectric power generation, Borçka Dam Lake is also influenced by intensive cage aquaculture operations, which represent another substantial anthropogenic pressure. Consequently, Borçka Dam Lake represents a freshwater system influenced by multiple anthropogenic pressures, including hydropower production and aquaculture (Emi̇nağaoğlu et al., 2016; Yıldırımer & Özalp, 2024).

Deriner Dam Lake, situated on the Çoruh River in Artvin province of northeastern Türkiye, is one of the nation’s most significant hydroelectric structures. Formed by a high double-curvature concrete arch dam, among the tallest in the country, the reservoir plays a central role in regional energy production by exploiting the steep gradients and strong flow conditions of the Çoruh Basin. Its impoundment alters downstream hydrology, sediment dynamics, and flow regulation, thereby shaping the reservoir’s physical and ecological characteristics. The system receives inputs from several tributaries and surface runoff channels draining steep mountain slopes. These inflows, influenced by the basin’s highly erodible geology, deliver fluctuating amounts of suspended sediments, organic matter, and geochemical constituents, affecting turbidity, nutrient levels, and sediment accumulation patterns. Although large-scale anthropogenic pressures are limited compared with other reservoirs in the region, localized land use, road construction, and small settlements still contribute human-derived pollutants to the catchment. The combination of its distinct geomorphology, relatively undisturbed environment, and strategic hydropower role makes Deriner Reservoir an important freshwater system with both environmental and socio-economic significance within the Çoruh River Basin (Ozcelik & Tuzlu, 2018; Özşeker, 2019).

Torul Dam Lake, situated along the Harşit Stream in Türkiye’s Gümüşhane province, serves as a key component of the regional hydroelectric network, regulating flow and contributing to energy production within the Harşit Basin. Formed by a concrete arch dam, the reservoir exhibits hydrological and morphological characteristics typical of steep, confined mountain valleys. Several small tributaries and surface runoff pathways discharge into the reservoir, transporting suspended sediments, organic material, and geochemical constituents, particularly during periods of high rainfall or snowmelt. In addition to these natural inputs, the Torul catchment is influenced by various anthropogenic pressures. Settlements located along the Harşit Valley, agricultural lands, transportation corridors, and small-scale industrial activities introduce pollutants through domestic wastewater, agricultural runoff, and erosion-driven inputs. These pressures can impact water quality, alter nutrient dynamics, and contribute to the accumulation of metals and other contaminants in the reservoir’s sediments. Another notable anthropogenic influence is the presence of aquaculture operations, particularly cage-based fish farming, which has become increasingly common in the region. These activities may contribute organic matter and nutrients to the reservoir system. The cumulative effects of these inputs may influence the ecological functioning of the reservoir and increase its susceptibility to eutrophication or other water quality impairments (Bayraktar et al., 2009, Eker 2020).

Taken together, these reservoir ecosystems offer a robust framework for examining the status and potential risk of metal contamination across seasons, reflecting diverse levels of human influence, ecological traits, and pollution pressures. In essence, the choice of these inland water bodies as study sites was driven by their ecological relevance, the extent of anthropogenic impacts, and their distinctive hydrological characteristics. These systems represent freshwater environments exposed to varying anthropogenic pressures.(Ozseker & Eruz, 2017; Özşeker et al., 2022a, 2022b).

### Field sampling, sample preparation

**Fig. 1:**
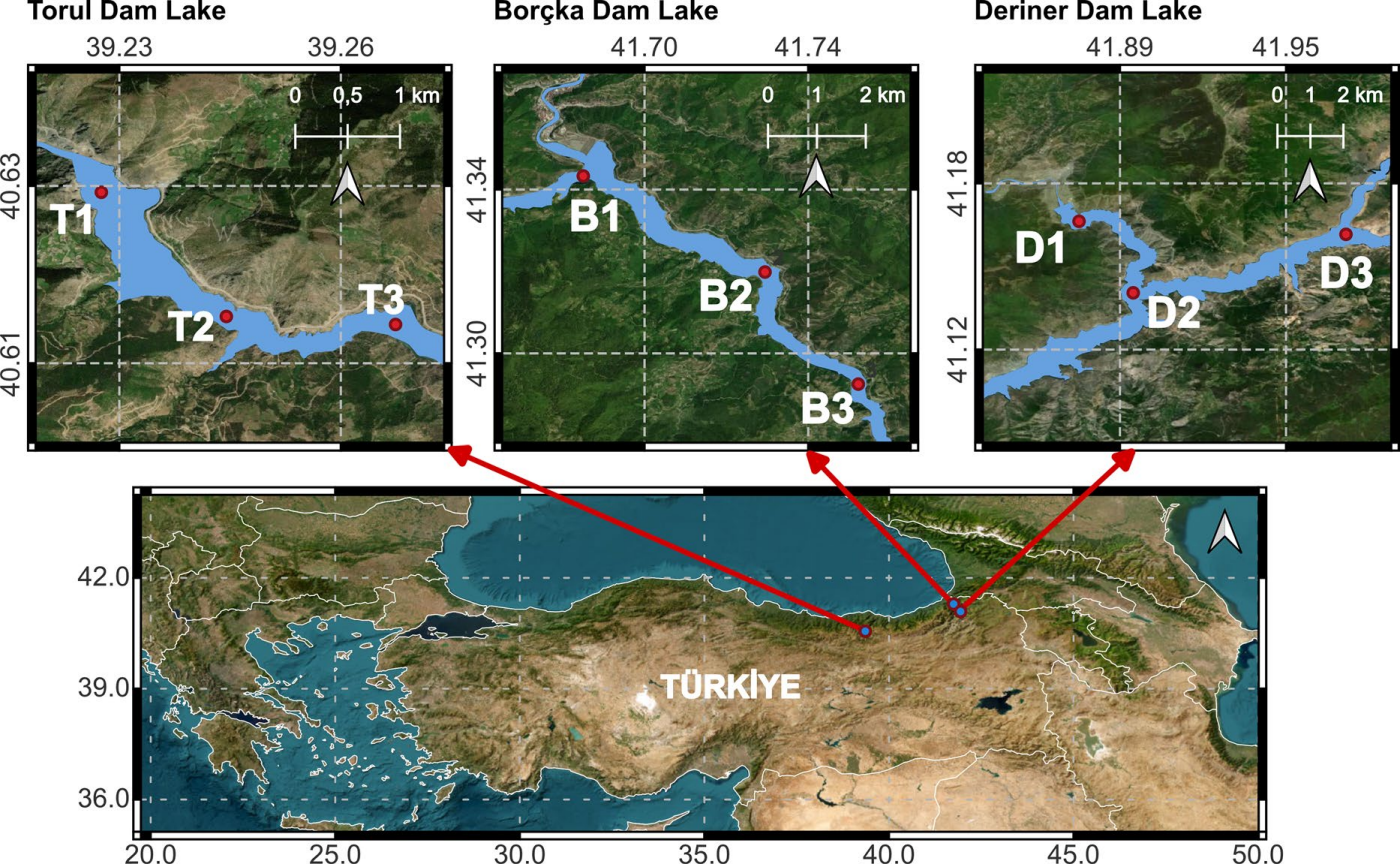
Location map of the reservoirs included in the study along with the identified sampling stations

This study aims to investigate the spatial and temporal dynamics of toxic metal pollution in different size fractions of surface sediments from the Borçka, Deriner, and Torul Dam Lakes, and to assess their potential ecological impacts using various pollution indices. In this context, seasonal sampling (i.e., spring (April), autumn (October) was conducted at three designated stations for 2024. In this context, seasonal sampling (i.e., spring (April) and autumn (October) was conducted in 2024 at three designated stations for each reservoir. Sampling locations were selected by considering the lake’s hydrodynamic patterns, including circulation and depositional areas, as well as zones likely to receive inputs from industrial, agricultural, and domestic activities. Taking these characteristics into account, three distinct stations were identified for each lake of each dam (Fig. 1).

Surface sediment samples were collected using an Ekman grab sampler. For the analyses, the upper 0–5 cm layer of each sediment sample was utilized (Özşeker & Terzi, 2025; Pelletier et al., 2022). The samples were then dried at 45 °C to obtain constant weight. To evaluate how grain size influences metal concentrations, the sediment samples were classified into four distinct size classes—0.063 mm (F1), 0.125 mm (F2), 0.25 mm (F3), and 0.5 mm (F4)—using standard sieve separation techniques. Particle fractionation was performed with the aid of a vibratory sieve shaker (AS200, Retsch, Duesseldorf, Germany), which ensured consistent and precise size-based segregation of the sediment material. (Gelhardt et al., 2021; Mattheus et al., 2020).

### Analytical methods and quality control

This research targets five key toxic metals, Copper (Cu), Lead (Pb), Zinc (Zn), Nickel (Ni), and Arsenic (As), which are naturally abundant in the region’s geological formations and warrant particular attention due to their potential ecological hazards (Radomirović et al., 2023). All sediment samples prepared for the determination of metal concentrations were analyzed using ICP-MS, a technique that offers high sensitivity for detecting metals at low concentrations and thereby enhances the analytical accuracy and reliability of the study (Resano et al., 2022; Wysocka, 2021). Prior to ICP-MS analysis, sediment samples were subjected to acid digestion using a microwave-assisted digestion system. Approximately 0.5 g of dried sediment was digested with a mixed acid solution consisting of HNO₃, HCl, and HF according to EPA Method 3052. Following digestion, the digested solutions were diluted with ultrapure water prior to instrumental analysis. Quality assurance and quality control (QA/QC) procedures included procedural blanks, duplicate analyses, and certified reference materials. Recovery rates for the analyzed metals ranged between 91 and 106%, indicating acceptable analytical performance.

The repeatability of the analyses was high, with the relative standard deviation (RSD) for replicate measurements remaining below 5%. Analytical accuracy and precision were confirmed by employing the certified reference material STD DS-7 (marine sediment; Analytical Reference Material, High Purity Standards, USA). The method’s detection limits were determined to be 0.1 µg/g for Cu, Pb, and Ni; 1 µg/g for Zn; 0.5 µg/g for As; and 0.01% for Al.

Physicochemical variables, such as pH, dissolved oxygen, turbidity, conductivity, and temperature, were measured on-site. The pH of the sediment was assessed using a Hach Lange meter in a 1:2 (w/v) sediment–water slurry, following the procedure outlined (Cifuentes et al., 2021). Measurements of temperature, turbidity, conductivity, and dissolved oxygen were obtained with a CTD multiparameter probe, in accordance with the protocol of Xiao et al. (2023). Total organic carbon (TOC) was quantified using a modified Walkley–Black wet oxidation method, which offers a detection limit below 0.05%, as described by Mustapha et al. (2023).

### Sediment quality guidelines

Within the Sediment Quality Guidelines (SQGs), the Threshold Effect Level (TEL) defines a contaminant concentration below which adverse biological effects are unlikely to occur (MacDonald et al., 2000; Smith et al., 1996). The Probable Effect Level (PEL), by contrast, represents a concentration above which toxic responses are expected with increasing frequency. The Effects Range Low (ERL) denotes the lower boundary at which measurable toxicity may begin to emerge, whereas the Effects Range Median (ERM) identifies concentrations associated with a substantially higher probability of ecological impairment (Al-Awah et al., 2026; Bhatt et al., 2025; Duman & Eronat, 2026; El-Sorogy et al., 2024).

SQGs provide a quantitative benchmark framework for evaluating sediment contamination and predicting biological risk. Concentrations below TEL and ERL thresholds are typically classified as low risk, while those approaching or exceeding PEL and ERM levels indicate a potential ecological effect on aquatic organisms.(Popoola et al., 2023; Wang et al., 2025; Xu et al., 2026). In this study, TEL, PEL, ERL, and ERM values were applied as reference criteria to characterize the ecological implications of metal contamination in reservoir sediments.

### Approaches to ecological and environmental risk evaluation

To evaluate the pollution status and ecological risk associated with metal accumulation in the reservoirs, five widely accepted geochemical and ecological indices were applied. The geoaccumulation index (Igeo) was used to quantify the degree of anthropogenic enrichment by comparing present metal concentrations with background levels after logarithmic scaling (Nasir et al., 2023). The enrichment factor (EF) was calculated to distinguish between natural and human-induced metal inputs through normalization against a conservative reference element (Jadoon et al., 2025). To assess the potential ecological threat posed by individual metals, the Ecological Risk Index (Er) was employed, incorporating toxic response coefficients and contamination factors to estimate the risk associated with each metal. In addition, the Potential Ecological Risk Index (PERI) was used to evaluate the cumulative ecological threat posed by multiple metals simultaneously (Al-Awah et al., 2026). Additionally, the Toxic Risk Index (TRI) was used to classify sediments based on their toxicological severity by integrating guideline-based concentration thresholds (Pelletier et al., 2022). These indices collectively provide a comprehensive assessment framework, enabling the identification of contamination sources, quantification of pollution intensity, and evaluation of potential ecological risks in the study area.

### Statistical methods and evaluation process

To assess whether metal concentrations differed significantly among the Borçka, Deriner, and Torul reservoirs, the non-parametric Kruskal–Wallis H test was employed (Clark et al., 2023). When significant overall differences were identified, Dunn’s post-hoc test was applied to determine the specific reservoir pairs responsible for the observed variation. P-values from the post-hoc comparisons were adjusted using appropriate multiple comparison correction procedures (e.g., Bonferroni or Holm) to control for Type I error (Agbangba et al., 2024).

The associations between metal concentrations and physicochemical parameters were examined using Spearman’s rank correlation, a robust non-parametric method suitable for non-normally distributed datasets and potentially non-linear relationships (Yu & Hutson, 2024).

To further investigate multivariate patterns among the reservoirs and to identify the principal axes underlying variation in metal concentrations, Principal Component Analysis (PCA) was conducted (Camargo, 2022). Prior to analysis, all variables were standardized (z-transformation) to remove scale-dependent effects. In addition, PERMANOVA (Permutational Multivariate Analysis of Variance) was applied to evaluate differences in the multivariate structure of metal concentrations among reservoirs (Garrido-Martín et al., 2023).

All statistical analyses were performed using the R statistical software environment (R Core Team, 2024), with relevant packages utilized for non-parametric testing, correlation analysis, multivariate statistics, and data visualization. (Table 1)

**Table 1:** Indices Applied for Ecological and Environmental Risk Assessment

Indices	Formulation	Class	Range	Remark
Sediment Enrichment Factor (SEF)	$SEF = \frac{{\left( {Ci/Cref} \right)Sample }}{{\left( {Ci/Cref} \right)Earths crust }}$	1	1–3	Minor enrichment
		2	3–5	Moderate enrichment
	Cf = Cd/Cr	3	5–10	Moderate-severe enrichment
		4	10–25	Severe enrichment
		5	25–50	Very severe enrichment
		6	> 50	Extremely severe enrichment
Ci is the content of metal; Cref is the content of reference, Cd is the content of metal; Cr is reference value of metal				
Geoaccumulation Index (Igeo)	Igeo = log_2_^[Cn∕(1.5Bn)]^	0	< 1	Uncontaminated
		1	0–1	Uncontaminated-Moderately
		2	1–2	Moderately
		3	2–3	Moderately-Heavily
		4	3–4	Heavily
		5	4–5	Heavily-Extremely
		6	> 5	Extremely
Cn is content of metal; Bn is background value of metal				
Ecological Risk Index (Er)/ Total Ecological Risk Index (RI)	CF = CD/CR	1	< 40/150	Low/Low
	ER = TR*CF			
	$$RI = \mathop \sum \limits_{i = 1}^{m} E_{r}^{i}$$			
		2	40–80/150–300	Moderate/Moderate
		3	80–160/300–600	Considerable/Considerable
		4	160–320/ > 600	High/Very high
		5	> 320	Very high
TR is the toxicity response coefficient of metal, they are 5, 5, 1, 5, and 10 for Cu, Pb, Zn, Ni, and As, respectively				
Toxic Risk Index (TRI)	$TRI = \sqrt {\frac{{(Ci/TEL)^{2} + (Ci/PEL)^{2} }}{2}}$ $\mathop {TRI = \sum }\limits_{i = 1}^{n} TRI$	1	≤ 5	No toxic risk
		2	5–10	Low toxic risk
		3	10–15	Moderate toxic risk
		4	15–20	Considerable toxic risk
		5	≥ 20	Very high toxic risk
Ci is the content of metal, TELis the threshold effect level, PEL is the probable effect level				

## Results

### Physicochemical profile and environmental characteristics

The seasonal (i.e., spring and autumn) evaluation of physicochemical parameters measured in the Borçka, Deriner, and Torul Dam Lakes provides important insights into the temporal variation of metal concentrations in sediment (Table 2). pH values remained within a consistently alkaline range (7.75–7.99) across all stations, indicating stable carbonate buffering and minimal risk of acidification, conditions that strongly influence metal speciation and sorption–desorption equilibria. Dissolved oxygen (DO) levels were markedly higher in spring (Borçka: 8.86–10.18 mg/L; Deriner: 9.02–10.08 mg/L; Torul: 9.35–10.38 mg/L), reflecting enhanced vertical mixing, cooler temperatures, and increased primary productivity. The decline in DO during autumn corresponded with thermal stratification and elevated microbial respiration, pointing to weakened mixing dynamics.

**Table 2:** Seasonal Dynamics of Key Physicochemical Parameters in the Study Area

Location	Season	Station	pH	Dissolved Oxygen (mg/L)	Temperature (°C)	TOC (mg/L)	Turbidity (NTU)	Conductivity (µS/cm)
Borçka Dam Lake	Spring	B1	7,81	10,18	12,2	6,61	20,8	384
		B2	7,99	9,49	12,6	5,02	16,8	365
		B3	7,88	8,86	10,2	3,38	11,6	370
	Autumun	B1	7,74	9,32	15,2	5,91	21,5	366
		B2	7,83	7,7	16,1	4,95	16,5	368
		B3	7,88	8,33	17,2	3,18	16,2	346
Deriner Dam Lake	Spring	D1	7,78	9,07	10,7	4,58	11,3	268
		D2	7,79	8,92	11,8	3,31	6,8	271
		D3	7,89	10,08	10,3	2,55	4,6	259
	Autumun	D1	7,97	7,84	17	4,38	10,3	280
		D2	7,84	7,74	15,9	3,12	10	275
		D3	7,94	8,59	16,5	2,44	7,2	273
Torul Dam Lake	Spring	T1	7,75	8,15	13,6	5,58	16,8	322
		T2	7,81	9,75	10,4	4,34	17,7	306
		T3	7,75	10,38	11,3	3,28	9,7	315
	Autumun	T1	7,86	7,93	17,1	5,12	15,6	304
		T2	7,94	8,39	16,2	4,11	10,5	307
		T3	7,91	8,35	15,1	3,31	15,7	310

Temperature exhibited the expected seasonal contrast, with low spring values and substantial autumn increases, highlighting the transition from mixing-dominated to stratified hydrodynamic conditions. This shift likely affects vertical particulate redistribution and the mobility of particle-bound metals. Total organic carbon (TOC) displayed reservoir-specific patterns: Borçka showed elevated TOC in spring followed by autumnal declines, consistent with runoff-driven allochthonous inputs transitioning to intensified microbial mineralization under warmer conditions. In contrast, Deriner and Torul exhibited more stable TOC levels, indicating relatively uniform organic matter dynamics.

Turbidity patterns reflect watershed-dependent sediment fluxes. Borçka consistently exhibited the highest turbidity (up to 21.5 NTU), driven by substantial sediment inputs from the mining-impacted Murgul Stream. Deriner presented moderate turbidity values, while Torul showed comparatively low and stable levels, suggesting more efficient sediment retention and reduced external sediment loading. Electrical conductivity (EC) exhibited limited seasonal fluctuation but notable spatial variation. Higher EC values in Borçka (up to 384 µS/cm) indicate elevated ionic loads, likely linked to geological and anthropogenic inputs. Lower EC values in Deriner and moderate levels in Torul reflect differences in freshwater inflow and mineralization patterns.

Overall, the seasonal shifts in physicochemical parameters highlight the dominant roles of hydrodynamic mixing, sediment inputs, and organic matter fluxes in shaping water-column conditions. These processes collectively influence suspended particulate behavior and govern the mobility of associated toxic metals, providing essential context for subsequent evaluations of metal distribution and ecological risk.

### Geochemical variability and statistical evaluation of metal levels in sedimentary environments

**Fig. 2:**
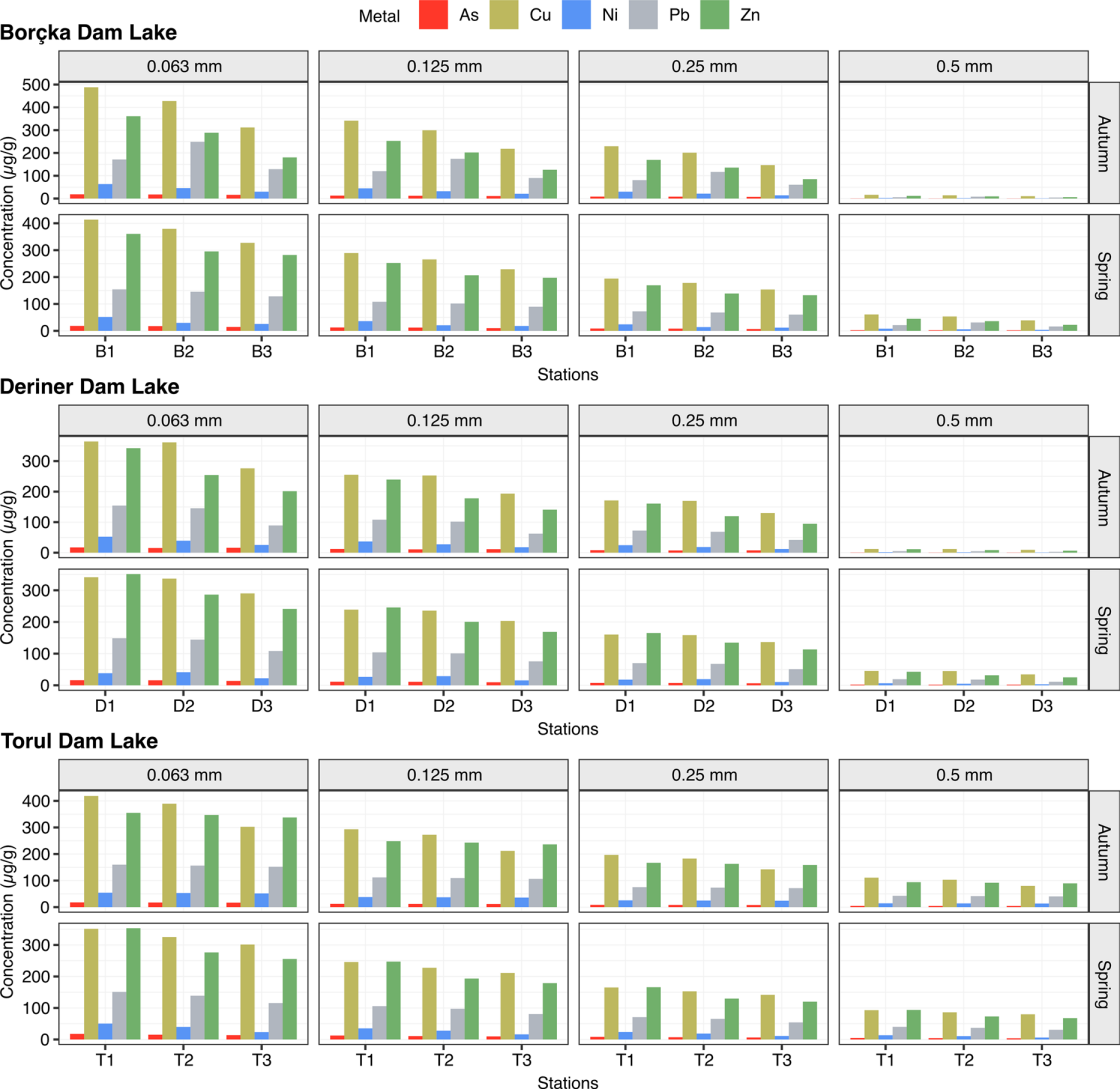
Seasonal (autumn–spring) patterns of metal distribution (As, Cu, Ni, Pb, Zn) across four sediment size fractions in Borçka, Deriner, and Torul Dam Lakes. Panels illustrate how metal levels vary simultaneously with grain size and sampling stations

Clear spatial and grain size dependent variations in toxic metal concentrations were observed among the reservoirs (Fig. 2). In the finest fraction (< 0.063 mm (F1)), metals exhibited their highest levels. During spring in Borçka Dam Lake, mean concentrations reached 373.3 ± 43.5 µg/g Cu, 142.6 ± 13.2 µg/g Pb, 312.5 ± 41.9 µg/g Zn, 35.5 ± 13.4 µg/g Ni, and 16.7 ± 2.1 µg/g As, increasing in autumn to 409.3 ± 89.5 µg/g Cu, 182.9 ± 60.8 µg/g Pb, 276.7 ± 90.8 µg/g Zn, 46.4 ± 16.9 µg/g Ni, and 17.3 ± 1.3 µg/g As. Deriner exhibited slightly lower but comparable values (e.g., 322.7 ± 28.3 µg/g Cu, 133.6 ± 22.1 µg/g Pb, 292.8 ± 55.3 µg/g Zn), while Torul presented the lowest spring levels (325.9 ± 24.9 µg/g Cu, 135.2 ± 18.1 µg/g Pb) but showed notable increases in autumn (370.2 ± 60.5 µg/g Cu, 156.1 ± 3.9 µg/g Pb) (Fig. 2).

In the 0.125 mm (F2) fraction, concentration decreased yet remained substantial. Spring means in Borçka were 261.3 ± 30.5 µg/g Cu, 99.8 ± 9.2 µg/g Pb, 218.7 ± 29.4 µg/g Zn, 24.9 ± 9.8 µg/g Ni, and 11.7 ± 1.4 µg/g As, rising in autumn to 286.5 ± 62.6 µg/g Cu, 128.1 ± 42.5 µg/g Pb, 193.7 ± 63.6 µg/g Zn, 32.5 ± 11.8 µg/g Ni, and 12.1 ± 0.9 µg/g As. Deriner and Torul followed similar patterns, although Torul exhibited more pronounced autumn increases, particularly for Zn (242.5 ± 6.0 µg/g) and Ni (37.1 ± 0.9 µg/g) (Fig. 2).

Metal concentrations declined further in the 0.25 mm (F3) fraction. Spring means in Borçka were 175.5 ± 20.5 µg/g Cu, 67.0 ± 6.2 µg/g Pb, and 146.9 ± 19.7 µg/g Zn, while Deriner recorded 151.7 ± 13.3 µg/g Cu, 62.8 ± 10.4 µg/g Pb, and 137.6 ± 26.0 µg/g Zn. Torul exhibited the lowest spring values but showed distinct increases in autumn, reaching 174.0 ± 28.4 µg/g Cu, 73.4 ± 1.8 µg/g Pb, and 162.8 ± 4.1 µg/g Zn (Fig. 2).

The coarsest fraction 0.5 mm (F4) contained the lowest metal levels across all reservoirs. Spring concentrations in Borçka averaged 98.9 ± 11.5 µg/g Cu, 37.8 ± 3.5 µg/g Pb, and 82.8 ± 11.1 µg/g Zn; Deriner and Torul exhibited similarly low values (e.g., Deriner: 85.5 ± 7.5 µg/g Cu, 35.4 ± 5.9 µg/g Pb; Torul: 86.4 ± 6.6 µg/g Cu, 35.8 ± 4.8 µg/g Pb). Autumn increases in this fraction were minimal and most apparent in Borçka (Fig. 2).

Overall, metal concentrations decreased systematically with increasing grain size (F1 > F2 > F3 > F4). Among the reservoirs, Borçka consistently showed the highest metal burdens, Deriner intermediate, and Torul the lowest concentrations across nearly all fractions. All reservoirs demonstrated a modest yet consistent increase in autumn, suggesting the influence of seasonal hydrological inputs and catchment-driven processes.

**Fig. 3:**
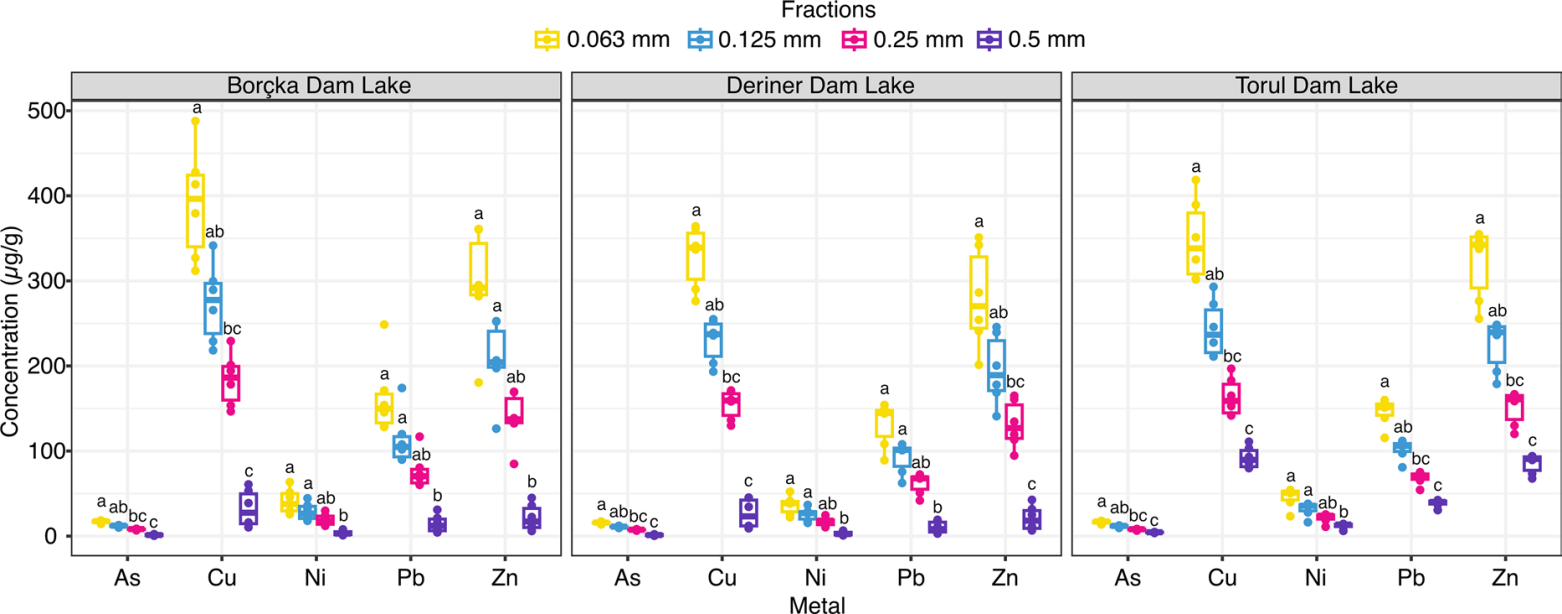
Metal concentrations in four sediment size fractions from Borçka, Deriner, and Torul Dam Lakes. Letters (a,b,c) denote statistically distinct groups based on post-hoc comparisons following ANOVA/Kruskal–Wallis testing (p < 0.05)

Marked and statistically robust differences in metal accumulation are evident among the four sediment size fractions (0.063 (F1), 0.125 (F2), 0.25 (F3), and 0.5 (F4) mm) across the Borçka, Deriner, and Torul Dam Lakes (Fig. 3). According to the variance-based statistical analyses, the finest fraction (0.063 mm) consistently exhibited the highest accumulation levels for all metals and was classified within the upper statistical group (a) across all lakes (p < 0.05). The 0.125 mm fraction generally represented intermediate accumulation patterns and was frequently grouped within the “ab” or “b” categories, indicating moderately but significantly lower levels compared with the finest fraction. A further decrease in accumulation was observed in the 0.25 mm fraction, which was commonly placed within lower statistical groups (b–c). The coarsest fraction (0.5 mm) exhibited the lowest accumulation levels among all fractions and was consistently assigned to the bottom statistical group (c). This hierarchical pattern, characterized by decreasing accumulation from the finest to the coarsest fractions, was highly consistent across all three lakes. The distribution of statistical grouping letters (a–c) clearly demonstrates that sediment grain size exerts a significant influence on metal partitioning and that at least two distinct statistical groups are formed for each metal. Overall, the results confirm that fine-grained fractions play a dominant role in metal retention and that fraction-dependent differences contribute substantially to the observed variability in sediment-associated metal concentrations.

**Fig. 4:**
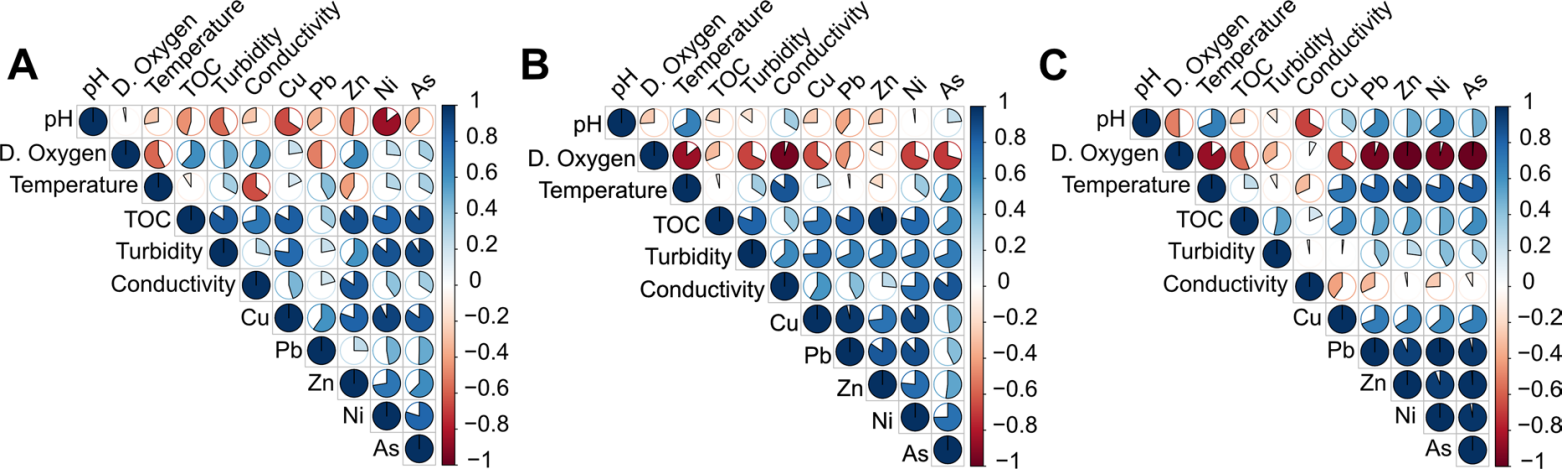
Correlograms showing the Pearson correlations between metals and physicochemical parameters in Borçka (A), Deriner (B), and Torul (C) Dam Lakes. The color and circle fill indicate the direction and strength of the correlations

Marked and coherent correlation patterns were observed between physicochemical variables and metal concentrations in the surface sediments of the three reservoirs (Fig. 4). In all lakes, metals were strongly and positively inter-correlated, forming a compact cluster that indicates common or co-varying behavior. Metal concentrations also showed consistently positive correlations with TOC, turbidity and conductivity, whereas their relationships with dissolved oxygen and pH were predominantly negative or weak. Temperature exhibited more lake-specific association patterns with both metals and water-quality variables. Overall, the correlograms highlight that metal enrichment in the sediments is closely linked to organic matter content and particle load, while more oxic and alkaline conditions tend to coincide with reduced metal levels.

**Fig. 5:**
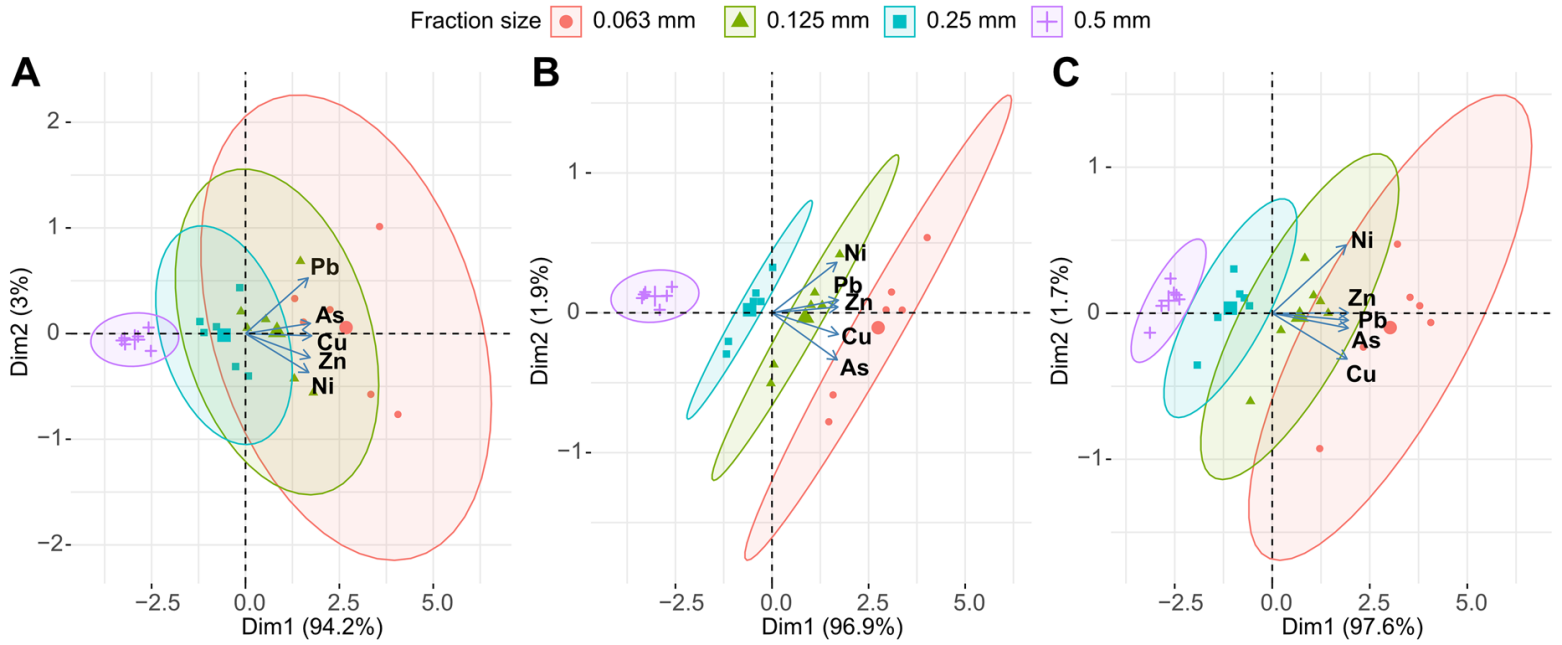
PCA ordination illustrates the separation of grain-size fractions in relation to metal loadings

PCA analysis conducted for all three dam lakes revealed a clear separation between sediment grain-size fractions and their associated metal concentrations (Fig. 5). For the Borçka Dam Lake, the first principal component explained 94.2% of the total variance, while the second component accounted for 3.0%, indicating that nearly all the variation originated from the differences among grain-size fractions. In the Deriner Dam Lake, Dim1 explained 96.9% and Dim2 explained 1.9% of the variance, with the finer fractions (0.063 and 0.125 mm) showing a stronger association with metal loadings and forming closely clustered groups within the PCA space. In the Torul Dam Lake, Dim1 accounted for 97.6% and Dim2 for 1.7% of the variance, similarly demonstrating that the fine fractions exhibited higher loadings along the direction of the metal component vectors (As, Cu, Pb, Zn, Ni). The consistent alignment of metal vectors across all lakes indicates a strong shared covariation among metals, while the marked separation among grain-size fractions highlights grain size as the primary factor shaping metal distribution patterns. Overall, the PCA results clearly show that metal accumulation is distinctly partitioned by sediment fractions in all lakes and that the majority of the variance (> 94%) is driven by these fraction-related differences. These PCA patterns were generally consistent with the correlation analysis results, indicating strong associations between fine-grained fractions, TOC, turbidity, and metal accumulation.

### Evaluation of sediment pollution indexes and quality guidelines

**Fig. 6:**
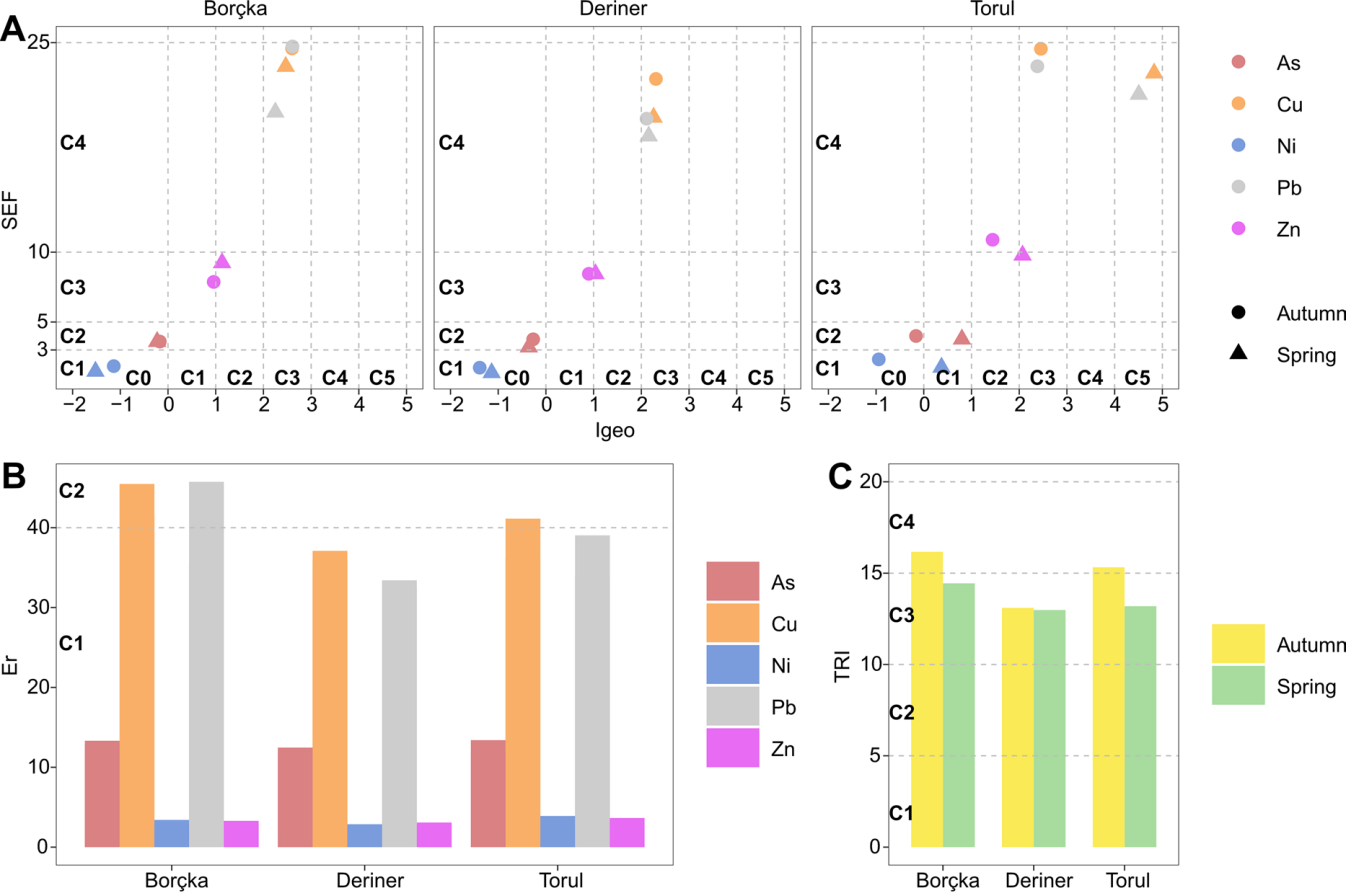
Classification-based evaluation of metal contamination and risk levels across the reservoirs studied. (A) Distribution of metals based on the relationship between the enrichment factor (SEF) and the geoaccumulation index (I geo). (B) Ecological risk (Er) classifications for individual metals, with class codes indicating low to moderate ecological risk levels. (C) Toxic Risk Index (TRI), illustrating reservoir-scale toxicity classifications. Here, the letter **C** denotes the class codes used to categorize contamination severity and toxic risk levels across the figure

A comprehensive evaluation of enrichment factors, geochemical indices, and ecological risk metrics revealed pronounced spatial and seasonal variability in metal contamination across the studied reservoirs. The classification of SEF and Igeo values (Fig. 6A) showed distinct element-specific enrichment and pollution patterns. For SEF, Cu and Pb predominantly fell within Class 4 (range 10–25; severe enrichment), whereas Zn consistently occupied Class 3 (range 5–10; moderate–severe enrichment). Ni remained within Class 1 (range 1–3; minor enrichment), and As within Class 2 (range 3–5; moderate enrichment). The Igeo results indicated that Cu and Pb were categorized into Class 3 (range 2–3; moderately–heavily polluted) for all reservoirs and seasons except in Torul during spring, where both metals reached Class 5 (range 4–5; heavily–extremely polluted), highlighting an episodic contamination peak. Zn exhibited Class 2 (range 1–2; moderately polluted) in Borçka and Deriner during spring and in Torul during autumn but shifted to Class 1 (range 0–1; uncontaminated–moderately polluted) in Borçka and Deriner during autumn. The highest Zn contamination similarly occurred in Torul during spring, where Zn reached Class 3 (range 2–3; moderately–heavily polluted). In contrast, Ni and As remained within Class 0 (range 0–1; uncontaminated) across all reservoirs and seasons except for Torul in spring, where both increased slightly to Class 1 (range 0–1; uncontaminated–moderately polluted). Ecological risk assessments (Fig. 6B) revealed that Cu presented Class 2 (range 40–80; moderate risk) only in Borçka during both seasons and in Torul during autumn, whereas all remaining reservoir–season combinations showed Class 1 (< 40; low risk). Pb similarly showed Class 2 (range 40–80; moderate risk) exclusively in Borçka during autumn, with Class 1 (< 40; low risk) elsewhere. Zn, Ni, and As were consistently assigned to Class 1 (< 40; low risk) in all reservoirs and seasons. TRI results demonstrated uniform toxicity levels, with all reservoirs falling within Class 3 (range 10–15; moderate toxic risk) (Fig. 6C). Overall, these findings indicate that despite episodic contamination peaks, particularly in Torul during spring, metal-induced ecological pressure in the reservoirs remains moderate yet non-negligible, warranting continued monitoring.

Across the four sediment fractions (0.063, 0.125, 0.25, ve 0.5 mm), metal concentrations exhibited a clear grain-size dependence, with markedly higher levels in finer sediments (Table 3). The 0.063 (F1) mm fraction represented the most contamination-sensitive component of the system. In this fraction, Cu (322–409 µg/g), Pb (109–156 µg/g), and Zn (266–347 µg/g) concentrations exceeded natural background levels by several fold and consistently surpassed both TEL and ERL thresholds, frequently rising above PEL values and, in some cases, approaching ERM benchmarks. Exceedance of these SQG thresholds indicates not only substantial metal enrichment but also a high likelihood of adverse biological effects, especially for sensitive benthic organisms. In contrast, Ni (34–53 µg/g) and As (15–17 µg/g) generally remained within the TEL–PEL range, suggesting comparatively lower ecological concern.

**Table 3:** Metal concentrations across sediment fractions in three reservoirs and their compliance with reference and SQG thresholds

Fraction	Reservoir	Season	Cu (μg/g)	Pb (μg/g)	Zn (μg/g)	Ni (μg/g)	As (μg/g)
0.063 mm (F1)	Borçka	Spring	373.3 ± 43.5	142.6 ± 13.1	312.5 ± 41.9	35.5 ± 13.9	16.6 ± 2
		Autumn	409.2 ± 89.4	108.9 ± 60.7	276.7 ± 90.8	46.4 ± 16.8	17.3 ± 1.3
	Deriner	Spring	322.7 ± 28.3	133.6 ± 22.1	292.8 ± 55.3	33.9 ± 10.3	15.2 ± 1.3
		Autumn	333.9 ± 49.9	129.6 ± 35.3	265.9 ± 71.1	38.9 ± 13.5	16.2 ± 0.9
	Torul	Spring	325.9 ± 24.9	135.2 ± 18.1	294.9 ± 51.4	37.9 ± 13.7	15.6 ± 2
		Autumn	370.1 ± 60.5	156.1 ± 3.9	346.5 ± 8.6	53 ± 1.3	17.4 ± 0.4
0.125 mm (F2)	Borçka	Spring	261.3 ± 30.5	99.8 ± 8.2	218.7 ± 29.4	24.9 ± 9.8	11.7 ± 1.4
		Autumn	286.5 ± 62.6	128 ± 42.5	193.7 ± 63.6	32.5 ± 11.8	12.1 ± 0.9
	Deriner	Spring	225.9 ± 19.8	93.5 ± 15.5	204.9 ± 38.7	23.7 ± 7.2	10.6 ± 0.9
		Autumn	233.7 ± 34.9	90.7 ± 24.7	186.1 ± 49.8	27.3 ± 9.4	11.3 ± 0.6
	Torul	Spring	228.2 ± 17.4	94.6 ± 12.6	206.5 ± 35.9	26.5 ± 9.6	10.9 ± 1.4
		Autumn	259.1 ± 42.3	109.3 ± 2.7	242.5 ± 6	37.1 ± 0.9	12.2 ± 0.3
0.25 mm (F3)	Borçka	Spring	175.5 ± 20.5	67 ± 6.2	146.8 ± 19.7	16.7 ± 6.5	7.8 ± 0.9
		Autumn	192.4 ± 42	85.9 ± 28.6	130.1 ± 42.7	21.8 ± 7.9	8.1 ± 0.6
	Deriner	Spring	151.7 ± 13.3	62.8 ± 10.4	137.6 ± 25.9	15.9 ± 4.8	7.1 ± 0.6
		Autumn	156.9 ± 23.5	60.9 ± 16.6	124.9 ± 33.4	18.3 ± 6.3	7.6 ± 0.4
	Torul	Spring	153.2 ± 11.7	63.5 ± 8.5	138.6 ± 24.1	17.8 ± 6.4	7.3 ± 0.9
		Autumn	173.9 ± 28.4	73.4 ± 1.8	162.9 ± 4	24.9 ± 0.6	8.2 ± 0.2
0.5 mm (F4)	Borçka	Spring	98.9 ± 11.5	37.8 ± 3.5	82.8 ± 11.1	9.4 ± 3.7	4.4 ± 0.5
		Autumn	108.5 ± 23.7	48.5 ± 16.1	73.3 ± 24.1	12.3 ± 4.5	4.6 ± 0.3
	Deriner	Spring	85.5 ± 7.5	35.4 ± 5.9	77.6 ± 14.7	8.9 ± 2.7	4 ± 0.3
		Autumn	88.4 ± 13.2	34.3 ± 9.4	70.5 ± 18.9	10.3 ± 3.6	4.3 ± 0.3
	Torul	Spring	86.4 ± 6.6	35.8 ± 4.8	78.2 ± 13.6	10 ± 3.6	4.1 ± 0.5
		Autumn	98 ± 16.1	41.4 ± 1.1	91.8 ± 2.3	14 ± 0.4	4.6 ± 0.1
Refeence Values (μg/g)		WASV	45	20	95	68	13
		CCV	55	12.5	70	75	1.8
		TRV	16	31	110	16	6
	SQG	ERM	390	110	270	50	85
		PEL	197	91.3	315	36	17
		ERL	70	35	120	30	33
		TEL	35.7	35	120	30	33

SQG (for fresh water) Sediment quality guideline; ERM: Effect range median, PEL: Probable effect level, ERL: Effect range low, TEL: Threshold effect level; WASV: worldwide average shale value, CCV: continental crust values, TRV: toxicity reference values

When evaluated across reservoirs, the magnitude of enrichment and SQG exceedance was most pronounced in Borçka Reservoir, where Cu and Pb concentrations regularly reached the upper end of the observed ranges and approached or surpassed PEL–ERM thresholds more frequently than in Deriner and Torul (Table 3). This pattern suggests a stronger and more persistent anthropogenic influence on metal accumulation in Borçka, indicating comparatively greater ecological concern in Borçka than in the other reservoirs. Deriner displayed intermediate risk levels, while Torul generally showed lower, but still notable, contamination, particularly for Zn. Overall, the fine-grained sediments of Borçka represent a key zone requiring closer attention in terms of potential toxicological implications.

## Discussions

The results of this study clearly indicate that the spatial and temporal distribution of heavy metals in the Borçka, Deriner, and Torul reservoirs is predominantly controlled by sediment grain-size characteristics, rather than by seasonal hydrodynamic variability or reservoir-specific environmental conditions. Metal concentrations showed a consistent decreasing trend from the finest fraction (< 0.063 mm) to the course (0.5 mm), confirming that fine-grained sediments act as the primary sinks for metal accumulation in lacustrine systems (Gloaguen et al., 2021; Subramanian et al., 2025).

This pattern can be attributed to the higher specific surface area, enhanced adsorption capacity, and stronger association with organic matter and mineral phases in finer particles, which facilitate the retention of metals. Beyond reflecting simple concentration differences, these findings demonstrate that sediment texture plays a fundamental role in controlling the partitioning, accumulation, and potential mobility of metals within reservoir environments. The consistent dominance of grain-size effects across multiple reservoirs and seasons further suggests that granulometric control represents a primary governing mechanism of metal distribution, potentially overriding other environmental drivers. Therefore, grain-size composition should be explicitly considered in sediment contamination assessments and ecological risk evaluations in freshwater systems.

Although fine-grained fractions exhibited the highest metal accumulation, interpretations based exclusively on these fractions should be approached cautiously, since the proportional contribution of fine particles to the total sediment mass may influence the overall ecological significance of contamination. Therefore, fraction-specific analyses should be evaluated together with bulk sediment characteristics to achieve a more balanced environmental assessment.

The exceptionally high concentrations of Cu, Pb, and Zn in the finest fraction, combined with PCA results indicating that more than 94% of the total variance is explained solely by grain-size segregation, underscore the pivotal role of fine-grained sediments, characterized by large surface areas, reactive mineral coatings, and elevated organic matter affinity, in promoting metal adsorption and accumulation (Vdović et al*.* 2021). These results not only align with established geochemical principles but also highlight the importance of integrating particle-size effects into ecological risk assessment frameworks, which have been largely overlooked in previous reservoir studies (Que et al*.* 2024).

Clear differences among the reservoirs reflect the combined influence of catchment geology, anthropogenic pressures, and sedimentological processes (Kaymak et al. 2023). Borçka Reservoir exhibited by far the highest metal burdens, a pattern directly attributable to the long-standing mining activities in the Murgul Basin, the high turbidity and suspended particulate loads transported by inflowing streams, and the organic enrichment associated with intensive cage aquaculture (Emi̇nağaoğlu et al., 2016, Şirin 2019, Yıldırımer & Özalp, 2024). The confined valley morphology further promotes fine-particle deposition, contributing to elevated Cu and Pb enrichment levels according to SEF and Igeo classifications (Ozseker & Eruz, 2017). In contrast, Deriner Reservoir displayed intermediate contamination levels, reflecting limited anthropogenic influence, stronger hydrodynamic mixing, and a more dispersed sediment texture that prevents the pronounced accumulation observed in Borçka (Özşeker, 2019). Torul Reservoir, while generally characterized by lower contamination levels, exhibited distinct autumnal peaks in Zn and Ni, likely linked to seasonal increases in runoff, agricultural and settlement-derived inputs, and localized organic matter enrichment from aquaculture activities (Bayraktar et al., 2009, Kodat and Tepe 2023). These episodic patterns highlight the sensitivity of mountainous reservoir systems to short-term environmental perturbations.

Seasonality contributed significantly to metal dynamics across all three reservoirs, with autumn consistently emerging as the period of highest metal accumulation. This trend appears to reflect enhanced catchment erosion during post-summer rainfall events, increased delivery of fine-grained particles, and the development of thermal stratification, which reduces dissolved oxygen concentrations in bottom waters and promotes the redox-mediated remobilization of previously bound metals (Naz et al*.* 2025, Liu et al., 2025). The pronounced seasonal increases observed in the finest fraction strengthen the argument that particle-size composition acts as both a driver and indicator of catchment disturbances. Such fine-scale responses would have remained undetected in traditional whole-sediment analyses, underscoring the necessity of fraction-specific assessments (Popovych et al*.* 2025).

The pollution indices and sediment quality guideline (SQG) evaluations provide additional insight into the ecological implications of the observed metal distributions (Ebrahimi Sarindizaj and Nikoo 2026, Shehu 2026). In Borçka, and to a lesser extent in Deriner, Cu and Pb reached enrichment and geoaccumulation levels corresponding to severe anthropogenic influence, reflecting the persistent impact of mining-derived inputs. The elevated Zn contamination observed in Torul during spring and autumn further emphasizes the episodic, flow-dependent nature of metal loading in this system. Notably, Cu, Pb, and Zn in the finest fraction frequently exceeded TEL and ERL thresholds and in several cases approached PEL and ERM benchmarks, indicating a tangible likelihood of toxic effects on benthic organisms (Zhang et al., 2022). Although Ni and As generally remained within low-risk ranges, the moderate overall toxicity indicated by TRI values across all reservoirs suggests that combined metal exposure may exert ecologically relevant stress(Eker and Kiliç 2024, Yang et al*.* 2024). These results collectively demonstrate that fine fractions serve as hotspots of ecological risk and should be prioritized in future monitoring and management programs.

A major contribution of this study lies in its novel integration of fraction-specific geochemical analysis, seasonal dynamics, and ecological risk indices within a multi-reservoir framework, representing the first comprehensive assessment of this kind conducted in Türkiye and one of the few globally to evaluate four grain-size fractions simultaneously. Previous studies have typically focused on bulk sediments, thereby obscuring fraction-specific metal accumulation patterns and limiting ecological interpretation associated with fine particulate matter.By explicitly linking grain-size variations with source processes, hydrodynamic controls, and guideline-based toxicity thresholds, this study provides a new conceptual model for understanding the physical and biogeochemical drivers of metal enrichment in reservoir ecosystems. The results therefore fill a critical gap in the literature and offer a refined methodological foundation for future sediment-based environmental assessments (Özşeker & Terzi, 2025). It should also be noted that different ecological risk indices and normalization approaches may yield partially different interpretations depending on the selected reference framework and weighting methodology.

Overall, the synthesis of fraction-resolved metal concentrations, seasonal hydrological dynamics, and ecological risk indicators highlights Borçka Reservoir as the system experiencing the greatest contamination pressure, driven by mining, particulate loading, and organic enrichment (Şirin 2019, Yıldırımer & Özalp, 2024). Deriner represents a comparatively less impacted yet environmentally significant system, whereas Torul exhibits lower baseline contamination but pronounced seasonal sensitivity, particularly with regard to Zn and Ni (Yegin and Karcioglu 2025). The demonstrated importance of fine-sediment fractions in shaping metal distribution and ecological risk underscores the need for fraction-specific monitoring and management strategies in reservoir systems (Przysucha et al. 2025, Xie et al*.* 2026). Consequently, this study not only advances scientific understanding of sediment–metal interactions but also provides actionable insights for the sustainable management of freshwater resources in regions facing similar environmental pressures.

Given that this study represents the first comprehensive investigation in Türkiye to examine fraction-specific metal accumulation in reservoir sediments across four distinct grain-size classes and two seasons, no national dataset is available for direct fraction-to-fraction comparison. Nevertheless, comparisons with both national and international studies on heavy metal contamination in lake ecosystems are presented in Table 4. The strong grain-size dependency observed in metal distributions in our findings is consistent with previous regional and global studies; however, the present research provides a more advanced methodological framework in terms of fraction diversity, seasonal resolution, and ecological risk assessment. For instance, Maslennikova et al. (2012) demonstrated that finer sediment fractions exhibit elevated metal concentrations in a lake/reservoir setting, a trend fully aligned with our results showing that Cu, Pb, and Zn reached their highest levels in the < 0.063 mm fraction. Yet, their study included a limited number of fractions and did not incorporate ecological risk indices, thus lacking a comprehensive evaluation of the environmental implications of metal enrichment. Similarly, research conducted in the Ruzín Reservoir (Ružičková et al. 2018) has shown that fine-grained sediments, particularly clay and silt, are enriched in Pb and Zn, paralleling our observations. However, those studies were typically restricted to a single reservoir and narrower analytical scope, with limited fraction resolution, no seasonal sampling, and the absence of integrated risk indices. Consequently, earlier studies were unable to fully interpret the ecological significance of pronounced increases in fine-fraction metal concentrations, such as the autumnal Zn and Ni peaks observed in Torul Reservoir. In contrast, the present study advances beyond the existing literature by combining multi-reservoir analysis, four grain-size fractions, seasonal sampling, and a suite of geochemical and ecological indices (Igeo, SEF, Er, TRI, and SQGs) (Zhang et al., 2022, Gutiérrez et al*.* 2023, Nasir et al., 2023, Bhatt et al., 2025, Shehu 2026). This integrated approach not only clarifies the mechanisms driving high Cu and Pb enrichment in Borçka due to mining inputs, the moderate contamination pattern in Deriner under limited anthropogenic influence, and the seasonally driven Zn and Ni fluctuations in Torul, but also provides a more robust geochemical and ecological interpretation than previous research, which addressed grain-size–metal relationships only at a generalized level.

**Table 4:** Comparative assessment of F1 fraction metal concentrations (Cu, Pb, Zn, Ni, As) in the present study and previously reported values from national and international lake and reservoir sediments

Location	Element					References
	Cu	Pb	Zn	Ni	As	
Present study (F1 Fraction)	391.3	125.8	294.6	40.9	16.9	Borçka Dam Lale
Present study (F1 Fraction)	328.3	131.6	272.9	36.4	15.7	Deriner Dam Lake
Present study (F1 Fraction)	348	145.6	320.7	45.5	16.5	Torul Dam Lake
Küçükçekmece Lake, Türkiye	67.7	30.2	183.9	92.8	9.2	(Kükrer et al., 2019)
Bafa Lake, Türkiye	26.80	12.30	36.33	174	n.a	(Algül & Beyhan, 2020)
Hazar Lake, Türkiye	55.2	18.1	87.8	126.7	19.6	(Varol et al., 2020)
Ömerli Dam Lake, Türkiye	43.9	40.7	231.2	73	9.5	(Güzel et al., 2022)
Beyşehir Lake, Türkiye	42.8	12	48.7	56.3	12.9	(Şener et al*.* 2023a)
Sera Lake, Türkiye	76.9	156.5	390	130.3	8.7	(Ozseker & Eruz, 2017)
Uzungöl Lake, Türkiye	307.2	155.9	258.8	24.7	8.5	(Ozseker & Eruz, 2017)
Borçka Dam Lake, Türkiye	446.5	154.6	356.3	56.6	17.8	(Ozseker & Eruz, 2017)
Suat Uğurlu Dam Lake, Türkiye	46	8.2	34	16	2.9	(Varol et al., 2022)
Saraydüzü Dam Lake, Türkiye	18	24	36	22	4.9	(Varol et al., 2022)
Ayvacık Dam Lake, Türkiye	7.8	5.2	15.5	13.6	n.a	(Tekiner et al., 2025)
Goplo Lake, Poland	14.8	19.4	87.3	6.9	2.1	(Juśkiewicz & Gierszewski, 2022)
Changsha Dam Lake, China	47.7	71.7	234.5	509.3	n.a	(Yang et al*.* 2026)
Wulungu Lake, China	10.6	13.2	59.8	12.4	2.7	(Liu et al., 2023)
Sochagota Lake, Colombia	104	21	85	53	36	(Cifuentes et al., 2021)
Kaptai Lake, Bangladesh	24.1	24.6	83.2	51.7	7.8	(Islam et al., 2023)
Ranwu Lake, Tibet	n.a	27.5	62.6	16.6	16.9	(Zhang et al., 2023)
Dongting Lake, China	37.7	53.5	163.8	34.3	n.a	(Chen et al*.* 2019)
Mariout Lake, Egypt	82.2	48.7	n.a	54	n.a	(Abu El-Magd et al., 2021)
Dongping Lake, China	36.9	26.4	76.1	39.5	19.1	(Zhang et al., 2022)
Uchalli Lake, Pakistan	12	7	45	n.a	4.2	(Aftab et al., 2023)
Michigan Lake, USA	54.3	95	260	37.5	10.3	(Christensen et al., 2025)
Mainit Lake, Philippines	64.9	45.2	152	37.3	18.9	(Laudiño et al., 2024)
Putheri Lake, India	12.1	5	16.3	21.1	n.a	(Thangaraj et al., 2025)
Wanfang Lake, China	10.3	11.5	31.7	5.11	1.86	(Chang et al., 2025)

n.a. not available

The F1 fraction metal concentrations measured in the present study, Cu (328.3–391.3 µg/g), Pb (125.8–145.6 µg/g), and Zn (272.9–320.7 µg/g), indicate comparatively elevated Cu, Pb, and Zn levels relative to many previously reported freshwater lake and reservoir systems. For instance, studies from Küçükçekmece (Cu: 67.7; Pb: 30.2; Zn: 183.9 µg/g), Hazar (Cu: 55.2; Pb: 18.1; Zn: 87.8 µg/g), Bafa (Cu: 26.8; Pb: 12.3; Zn: 36.3 µg/g), Ömerli (Cu: 43.9; Pb: 40.7; Zn: 231.2 µg/g), and Beyşehir lakes (Cu: 42.8; Pb: 12; Zn: 48.7 µg/g) consistently reveal substantially lower concentrations (Algül & Beyhan, 2020; Güzel et al., 2022; Kükrer et al., 2019; Şener et al., 2023; Varol et al., 2020). Similar trends appear at the international scale, where reported values from Goplo Lake, Poland (Cu: 14.8; Pb: 19.4; Zn: 87.3 µg/g), Uchalli Lake, Pakistan (Cu: 12; Pb: 7; Zn: 45 µg/g), Wulungu Lake, China (Cu: 10.6; Pb: 13.2; Zn: 59.8 µg/g), and Kaptai Lake, Bangladesh (Cu: 24.1; Pb: 24.6; Zn: 83.2 µg/g) fall well below the concentrations observed in this study (Islam et al., 2023; Juśkiewicz & Gierszewski, 2022; Liu et al., 2023). Only a limited number of systems, such as Sera Lake (Pb: 156.5; Zn: 390 µg/g) and Uzungöl (Cu: 307.2; Pb: 155.9; Zn: 258.8 µg/g), exhibit comparably elevated levels; however, even these values remain generally lower than the upper range documented here (Ozseker & Eruz, 2017). Conversely, Ni (36.4–45.5 µg/g) and As (15.7–16.9 µg/g) concentrations align more closely with mid-to-upper ranges reported globally. Overall, the elevated Cu, Pb, and Zn levels observed in the F1 fraction suggest notable metal accumulation within the studied reservoirs compared with many freshwater systems reported in the literature.

## Conclusions

This study demonstrates that toxic metal accumulation across different sediment size fractions in the Borçka, Deriner, and Torul reservoirs poses a substantial ecological concern for these interconnected freshwater ecosystems. The markedly elevated concentrations of Cu, Pb, Zn, Ni, and As in the fine fractions, particularly those < 63 µm, clearly indicate that these particles represent not only the primary carriers of metals but also the most ecologically sensitive and potentially bioavailable component of the sediment matrix. Spatial patterns reveal that areas influenced by riverine inputs and catchment-derived materials exhibit enhanced metal loading, while seasonal variability reflects the strong coupling between hydrodynamic processes and the redistribution of contaminants within the system. Comparative evaluation with national and international lake and reservoir environments confirms that the concentrations recorded here, especially for Cu, Pb, and Zn, are among the highest reported to date, suggesting elevated metal accumulation potentially influenced by anthropogenic activities within the region. Such elevated contaminant levels highlight the potential for adverse impacts on benthic communities, food-web dynamics, and long-term ecosystem functioning.

These findings indicate that bulk sediment concentrations alone may not fully reflect fraction-specific metal enrichment patterns and associated ecological implications, while fraction-specific analyses provide additional insight into contaminant mobility and biological availability. To safeguard ecological integrity, it is essential to implement continuous, grain-size-resolved monitoring programs, develop watershed-scale strategies to reduce pollutant inputs, and identify and manage potential contamination sources more effectively. Overall, this study emphasizes the urgent need for science-based environmental management and targeted mitigation actions to protect and sustain the ecological health of these reservoir ecosystems.

## Data Availability

No datasets were generated or analysed during the current study.
